# Frictionless Mechanics for Correction of Bimaxillary Protrusion: A Case Report

**DOI:** 10.7759/cureus.56522

**Published:** 2024-03-20

**Authors:** Srushti Atole, Ranjit Kamble, Sumukh Nerurkar, Japneet Kaiser

**Affiliations:** 1 Department of Orthodontics and Dentofacial Orthopedics, Sharad Pawar Dental College And Hospital, Datta Meghe Institute of Higher Education and Research, Wardha, IND

**Keywords:** extraction, frictionless mechanics, friction mechanics, reverse closing loop, bimaxillary protrusion

## Abstract

The condition of bimaxillary protrusion is commonly seen by orthodontic practitioners in the course of our daily clinical work. It is characterized by both jaws being forwardly placed and increased prominence of the teeth along with lips. When there is a severe bimaxillary protrusion, it can be challenging to correct it effectively with maximum anchorage. In patients with protrusions or crowding, extraction therapy is often necessary. There are two ways to retract anteriors during extraction space closure: friction or frictionless. The present case report explains the frictionless mechanic protocol for the correction of bimaxillary protrusion using a reverse closing loop.

## Introduction

Bimaxillary protrusion in orthodontics refers to a condition where both the upper and lower jaws protrude forward excessively, leading to prominent or protrusive lips and an increased prominence of the teeth [1]. This condition can affect both dental aesthetics and functionality, often causing concerns related to appearance and bite alignment. It is most common among African-American [2-3] and Asian [4-5] populations, but it can occur in almost all ethnic groups. Several studies have suggested that the prevalence of bimaxillary protrusion in India ranges from 8% to 25%, varying among different ethnic groups and geographical locations within the country.

The complex etiology of bimaxillary protrusion constitutes contextual influences such as lip-biting, the thrusting of the tongue, a larger volume of tongue and mouth breathing, along with the hereditary component [1]. The tongue is one of the key reasons for bimaxillary protrusion of incisors, as it exerts pressure on the dentition, which is not counteracted by the labial pressure, hence causing proclination of the incisors. Also, the habit of tongue thrusting applies extensive forces on the incisors, causing a similar problem. Whereas lip biting of the lower lip exerts pressure on the upper incisor, causing its proclination. Similarly, all the causes involving the tongue or habits are the environmental cause/etiology for bimaxillary protrusion. Orthodontic treatment plays a crucial role in managing bimaxillary protrusion. The goal of treatment is to minimize soft tissue procumbence and convexity by retroclinating and retracting the mandibular and maxillary incisors. [1]. Treatment involves removing the first premolars, and then anteriors retracted with maximal anchorage. Friction (sliding) mechanics or frictionless (loop) mechanics can be used to retract anterior teeth during extraction space closure [6]. Frictionless mechanics uses a wide variety of loops, including a vertical loop, reverse loop, boot loop, teardrop loop, T loop, omega loop, delta loop, mushroom loop, etc. [7]. Reverse loop was introduced by Dr. RH Strang in 1933 [8]. Reverse closing loop is relatively cheap, simple, easy to make, and easy to operate. The application of frictionless mechanics with a reverse loop in a bimaxillary protrusion situation is described in this case study.

## Case presentation

**Figure 1:**
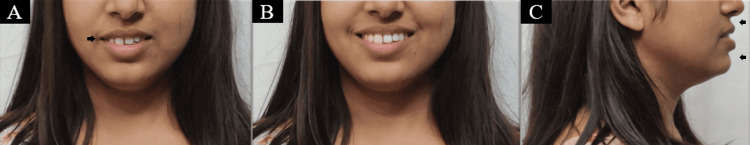
Extraoral pretreatment photographs: (A) frontal; (B) smiling; (C) profile

A female patient, aged 22, presented herself to the Department of Orthodontics 14 months back with the chief complaint of upper and lower front teeth being positioned forward. A convex profile was noticed during extraoral examination along with mesocephalic head form, mesoprosopic face form, incompetent lips, and bilaterally symmetrical face [9]. The patient had a full maxillary incisor display and a symmetrical, consonant smile with an acute nasolabial angle and a deep mentolabial sulcus (Figure 1).

**Figure 2:**
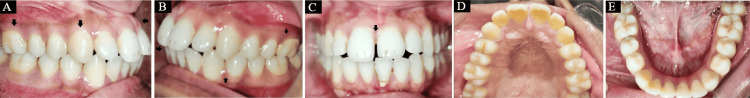
Intraoral pre-treatment photographs: (A) right side view of buccal occlusion; (B) left side view of buccal occlusion; (C) frontal labial; (D) occlusal surface of the maxillary arch; (E) occlusal surface of the mandibular arch

The intraoral examination revealed a normal maxillary and mandibular alveolar ridge, a normal tongue, a normal labial and buccal vestibule, and a healthy normal gingiva. Hard tissue examination revealed that all permanent teeth were present, with 3-mm overjet and 1-mm overbite. Both maxillary and mandibular anterior were proclined with midline diastema in the upper anterior and mild crowding in the lower arch. Both the right and left molar and canine relationship were class I (Figure 2).

**Figure 3:**
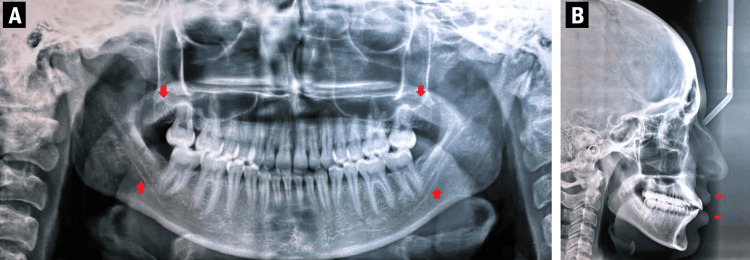
(A) Orthopantomogram and (B) lateral cephalogram

An orthopantomogram (OPG) examination revealed all teeth were present except third molars in all quadrants Figure 3A. According to cephalometric analysis, the patient was in cervical vertebral maturation index (CVMI) stage VI (completion) with class I skeletal bases and vertical growth patterns (sella-nasion (SN)-mandibular plane angle: 34°). She had proclined upper (1 to NA angle: 38°; 1 to NA mm: 10 mm) and lower incisors (1 to NB angle: 37°; 1 to NB mm: 9 mm). On soft tissue analysis, it was found that protrusive upper (9-mm prominence) and lower (8-mm prominence) lips. An acute nasolabial angle (70°) was found (Figure 3B).

Treatment plan

The treatment objective was to achieve the correct inclination of the upper and lower anterior to attain normal overbite and overjet, to close pre-existing spacing, and to maintain the class I molar and canine relationship. After the patient's motivation and education, the treatment plan includes bonding of both maxillary and mandibular arches and extraction of the first premolars of all four quadrants to correct proclination and prominence of lips. After extraction, initial leveling and alignment were planned, followed by the closure of the extraction space using frictionless mechanics. In the maxilla, transpalatal arch, mandible, and lingual arch were planned to enhance anchorage.

Treatment progress

**Figure 4:**
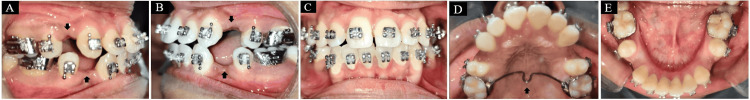
Intraoral stage photographs: (A) after initial leveling and alignment; (B) right lateral; (C) left lateral frontal view; (D) maxillary occlusal view depicting transpalatal arch; (E) mandibular occlusal view

The case was started by bonding both maxillary and mandibular arches using a McLaughlin, Bennet, and Trevisi (MBT) 0.022" slot prescription. After bonding, all quadrants’ first premolars were extracted. The transpalatal arch was cemented in the maxillary arch to enhance anchorage (Figure 4D). Initial leveling and alignment were practiced with a suitable archwires sequence, which includes 0.016" nickel-titanium (NiTi) wire, 0.016 × 0.022" NiTi wire, and 0.017 × 0.025" stainless steel (SS) wire [10] (Figure 4).

**Figure 5:**
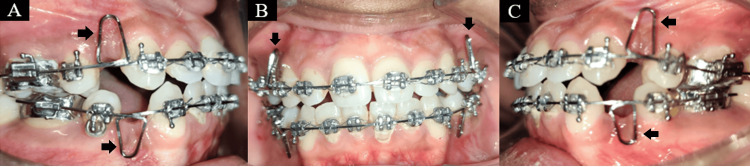
Intraoral photographs with reverse closing loop: (A) right side view of buccal occlusion; (B) frontal labial; (C) left side view of buccal occlusion

To prevent more anterior proclination, the archwires were cinched distal to the molar. Using 0.019 × 0.025" titanium molybdenum alloy (TMA) wire, the reverse closing loop was used for exhibiting en masse retraction; 15° of alpha while 25° of beta bends were given in a loop (Figure 5).

**Figure 6:**
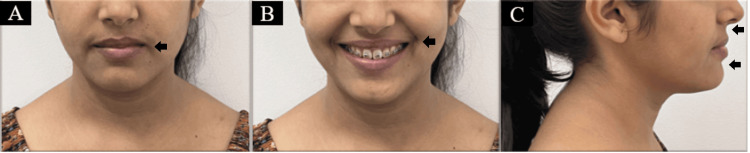
Extraoral current photographs: (A) frontal view depicting competent lips; (B) smiling frontal view; (C) profile view depicting average nasolabial angle and mentolabial sulcus

**Figure 7:**
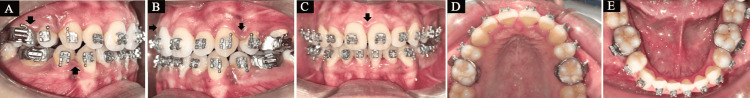
Intraoral current photographs depicting well-aligned arches with complete extraction space closure and maintained class I canine and molar relation: (A) right side view of buccal occlusion; (B) left side view of buccal occlusion; (C) frontal labial; (D) occlusal surface of the maxillary arch; (E) occlusal surface of the mandibular arch

The loop was activated by 2 mm every 1½ months with tight cinching back distal to molar. Retraction and complete extraction space closure were achieved within 14 months. Currently, both arches are well aligned with complete extraction space closure (Figures 6-7).

At present, arch wires with finishing bends are given for finishing and detailing of the occlusion. To avoid relapse, the permanent lingual retainer is planned immediately after debonding [11].

## Discussion

Bimaxillary proclination is a condition in which the anteriors of both arches are severally proclined, and lips have more prominence than normal. This type of malocclusion is commonly found in the Asian group of the population. Kocadereli et al. state that when a decrease in lip prominence is one of the objectives of planned treatment, extraction of premolars and incisors retraction is a suitable option to achieve this objective. That is why, based on the patient's chief complaint and the diagnosis of the malocclusion, to correct the proclination of anteriors, achieve lip competency, and decrease facial convexity, extraction of first premolars was carried out in the present case. Drobocky found that patients who had their first premolars extracted experienced an average reduction in lip prominence of 3.4 and 3.6 mm with Ricketts’ E-line [12].

The closure of the extraction space must be taken into consideration in the treatment plan when the malocclusion is corrected by extracting the premolars. The extraction sites can be closed by retraction of the anterior segments, protraction of the posterior segments, or both. Because mesialization of posteriors can compromise anterior tooth retraction, orthodontists are mainly concerned with anchorage maintenance. Nanda et al. [13] state that A group A anchorage refers to the conservation of the tooth position in the posterior, with more than 75% of space required for the retraction of anteriors. A number of auxiliaries, such as transpalatal arch, Nance holding button, temporary anchorage devices (TADs), frictionless method, or extraoral traction, are typically required to enhance the anchorage. According to Andreasen [14], retraction combined with adjunctive appliances controlled anchorage to a range of 0 to 2.4 mm in mesial molars. To augment anchorage in this patient, the transpalatal arch recommended by Goshgerian [15] was used; it is inexpensive, easy to fabricate, and most reliable.

In orthodontics, two types of mechanical mechanisms can close spaces, "sliding mechanics" is the first type, in which brackets and tubes slide over a wire. The alternative is "frictionless mechanics," in which the tooth or set of teeth moves as a result of the moment-to-force ratio generated during loop activation. In friction/sliding mechanics, 0.018 × 0.025-in archwire is used, while 0.019 × 0.025-in archwire in frictionless/loops mechanics. Compared to sliding mechanics, loop mechanics permits the use of a larger archwire size due to less friction and binding. During upper anterior teeth retraction, a larger wire size is more favorable for generating lingual root torque. For space closure, Faulkner, Burstone, and Germane recommended the utilization of loop mechanics for better torque control of anterior teeth [16-20]. Reverse loop was introduced by Dr. RH Strang in 1933. The reverse closing loop is simple, inexpensive, easy to construct, and simple to execute. It has been considered an excellent method of closing extraction spaces. Therefore, a reverse closing loop is chosen in the present case. It produces more rapid retraction with less tipping of anteriors.

## Conclusions

The utilization of a reverse closing loop in the treatment of bimaxillary protrusion presents a promising and effective orthodontic approach. Additionally, the use of the reverse closing loop appeared to provide advantages such as efficient tooth movement, reduced treatment duration, and minimized patient discomfort. A reverse closing loop produces more rapid retraction with less tipping of the canines. This is the major advantageous property of the reverse closing loop over the other retraction mechanics. Along with several advantages, there are some limitations to reverse closing the loop. The disadvantage of this loop includes the variations in the depth of the buccal sulcus, which may limit the loop's height.

In this case report, the application of the reverse closing loop demonstrated significant improvements in the correction of excessive dental protrusion and achieving desirable facial esthetics. The close monitoring and adjustments throughout the treatment ensured optimal progress and outcomes.
